# Phylogenetic Identity, Cyanotoxin Production Potential, and Thermal Performance in Native and Alien Nostocalean Cyanobacteria Under Climate Warming

**DOI:** 10.3390/biology15151316

**Published:** 2026-08-06

**Authors:** Izabelė Šuikaitė, Karina Šmeliova, Jūratė Karosienė, Daiva Burokienė, Dovilė Čepukoit, Judita Koreivienė

**Affiliations:** 1 Laboratory of Algology and Microorganisms Ecology, State Scientific Research Institute Nature Research Centre, Akademijos St. 2, LT-08412 Vilnius, Lithuania; karina.smel@outlook.com (K.Š.); jurate.karosiene@gamtc.lt (J.K.); 2 Laboratory of Plant Pathology, State Scientific Research Institute Nature Research Centre, Akademijos St. 2, LT-08412 Vilnius, Lithuania; daiva.burokiene@gamtc.lt (D.B.); dovile.cepukoit@gamtc.lt (D.Č.)

**Keywords:** microbial invasion, warming, thermotolerance, alien species, harmful blooms, disturbed ecosystems, freshwaters

## Abstract

Climate-driven warming may enhance the spread of warm-adapted alien cyanobacteria in freshwater ecosystems. This study assessed four nostocalean cyanobacteria from European lakes—native *Aphanizomenon gracile* and alien *Chrysosporum bergii*, *Raphidiopsis raciborskii*, and *Sphaerospermopsis aphanizomenoides*—to evaluate species identity, toxin production potential, and thermal responses. A polyphasic approach combining phylogenetic analysis, morphology, and toxin screening confirmed the identifications and revealed a saxitoxin-producing *A. gracile* strain. No toxin genes were detected in the alien taxa. Experimental testing across 15–35 °C demonstrated broader thermal tolerance in the alien species, which maintained positive growth across 20–35 °C but showed limited performance at 15 °C. In contrast, the native *Aphanizomenon gracile* maintained positive growth at 15 °C, indicating greater adaptation to cooler conditions. Isolate-specific variation at thermal extremes highlighted the importance of ecotype-level traits. Our findings suggest that rising temperatures, combined with strain-level physiological diversity, may increase the risk of alien cyanobacteria establishing and forming blooms in temperate lakes. This study supports the need for monitoring strategies that integrate taxonomy, ecophysiology, and local adaptation to better assess invasion potential under changing environmental conditions.

## Simple Summary

As the climate warms, some cyanobacteria (also known as blue-green algae) that normally thrive in warmer regions may spread into freshwater lakes in cooler parts of Europe. Some of these species can form harmful blooms that threaten water quality, aquatic life, and human health. In this study, we examined four cyanobacterial species found in European lakes: one native species and three alien species. We confirmed their identity, assessed their ability to produce toxins, and investigated how they respond to different water temperatures. The native species was better adapted to cooler water, whereas the alien species tolerated a broader range of temperatures but grew poorly at lower temperatures. Among the species studied, only the native species was able to produce a toxin. Our findings suggest that temperature rise may promote the spread and establishment of alien cyanobacteria in temperate lakes. Understanding their response to warming will help improve monitoring of harmful blooms.

## 1. Introduction

Cyanobacteria are an ancient prokaryote that originated around 3.5 billion years ago; therefore, these organisms demonstrate remarkable phylogenetic diversity, which has contributed to their adaptability across a wide range of environmental conditions and their widespread distribution in various habitats, including freshwater ecosystems. Under certain environmental conditions, cyanobacteria can form harmful algal blooms characterized by decline in biodiversity and overall ecosystem health [1,2,3]. In many cases, these impacts are exacerbated by the production of secondary metabolites, including cyanotoxins, which can influence phytoplankton succession and interspecific competition, and pose risks to animal and human health. Importantly, cyanotoxin production is highly variable and depends on species identity, strain-level genetic traits, and environmental conditions, rather than being a universal feature of bloom-forming cyanobacteria [4].

Among freshwater cyanobacteria, members of the order Nostocales are of particular ecological interest due to their capacity to form harmful blooms [1,5,6,7]. Several nostocalean species, including *Chrysosporum bergii*, *Raphidiopsis raciborskii*, and *Sphaerospermopsis aphanizomenoides*, are considered alien in Europe and have expanded beyond their native ranges into temperate freshwater ecosystems, with rising water temperatures as a possible factor for their distribution [1,5,8,9,10,11]. However, within the northern border of their spread in European lakes, water temperatures have been suggested to be insufficient for their rapid proliferation and bloom formation [12]. In contrast, the primary native nostocalean competitor for these alien cyanobacteria, *Aphanizomenon gracile*, has demonstrated the ability to form blooms under the same conditions [13]. Experimental studies suggest that alien nostocalean species may gain a competitive advantage over native taxa at elevated temperatures, although the research has only been conducted on a few strains [14,15].

Ongoing climate warming is expected to modify the thermal regimes of temperate freshwater ecosystems, with projected increases in both mean and extreme summer water temperatures across Europe [16]. Such changes are likely to reduce thermal constraints that currently limit the proliferation of warm-adapted cyanobacteria at higher latitudes, potentially facilitating the establishment and expansion of alien nostocalean species [6]. In addition to gradual warming, the increasing frequency and intensity of heatwaves may expose freshwater cyanobacterial communities to short-term temperature extremes that exceed historical norms [17,18]. Together, these shifts are expected to alter species interactions, competitive hierarchies, and bloom dynamics, underscoring the need to understand species- and strain-specific thermal responses under present and future climatic conditions.

Native *Aphanizomenon gracile* and alien *Chrysosporum bergii*, *Raphidiopsis raciborskii*, and *Sphaerospermopsis aphanizomenoides* form a phylogenetically heterogeneous group defined by distinct ecological characteristics. Moreover, the variability in physiological responses among different strains within these species provides insights for taxonomic differentiation and plays a crucial role in their ecological competitiveness, adaptability to new environments and invasion success [19]. Intraspecific variability in thermal responses has been documented in alien nostocalean species and may contribute to their adaptability under changing environmental conditions [20,21]. Nevertheless, comprehensive comparative data across multiple nostocalean species and strains remain scarce.

In this study, we investigated native and alien nostocalean cyanobacteria isolated from European freshwater ecosystems to (i) verify species identity using phylogenetic analysis of the partial PC-IGS region, (ii) evaluate their cyanotoxin production potential, and (iii) assess species- and strain-level growth responses across a broad temperature gradient. A polyphasic framework was applied at the species level, integrating phylogenetic validation with strain-level toxicological and physiological analyses. This integrative approach allows species-specific and intraspecific adaptive traits to be assessed in an ecological context, providing insight into how freshwater warming may alter competitive interactions between native and alien cyanobacteria, influence their establishment, and modify the risk of potentially toxic blooms in temperate freshwater ecosystems.

## 2. Materials and Methods

### 2.1. Cyanobacteria Isolation and Maintenance

The isolates of *Aphanizomenon gracile*, *Chrysosporum bergii*, *Raphidiopsis raciborskii*, and *Sphaerospermopsis aphanizomenoides* used in this study, along with their origin, year of isolation, habitat conditions, and the applied analyses (phylogeny, cyanotoxin screening, thermotolerance), are detailed in Table 1. In total, 39 isolates were obtained from the freshwater lakes Jieznas (54.592701° N, 24.180396° E), Gauštvinis (55.646733° N, 23.192962° E), Gineitiškės (54.737955° N, 25.185321° E), Rėkyva (55.865286° N, 23.301316° E), and Simnas (54.399536° N, 23.638337° E) in Lithuania; Hostivař Reservoir (50.039006° N, 14.540039° E) in the Czech Republic; and Lakes Witobelskie (52.265278° N, 16.725556° E) and Pniewskie (52.511389° N, 16.240833° E) in Poland during summer. The single filament isolates were retrieved from the lake water samples using a glass microcapillary pipette and grown in modified MWC medium [22] and maintained in a controlled growth chamber at illumination of 50 μmol photons m^−2^ s^−1^ under a 16:8 light:dark photoperiod regime at 20 °C. All isolates were identified based on their morphological characteristics, following the descriptions provided by Komárek [23] and deposited in the culture collection of Laboratory Algology and Microbial Ecology at State Scientific Research Institute Nature Research Centre (Lithuania).

### 2.2. Genomic DNA Extraction and PCR Amplification

About 40 mL of each 24 cyanobacterial isolates were centrifuged at 8000 rpm for 10 min and stored at −20 °C prior to analysis for cyanotoxin screening. Genomic DNA was extracted using the cetyltrimethylammonium bromide (CTAB) method following [26]. Polymerase chain reaction (PCR) was used to amplify the phycocyanin intergenic spacer (PC-IGS) region for phylogenetic analysis and to screen for genes associated with cyanotoxin biosynthesis (*cyrA*, *cyrJ*, and *sxtA*). The PC-IGS region was amplified using primers PCbF and PCaR according to [27]. The *cyrA* gene was amplified using primers cynsulF and cylnamR [28,29], and the *cyrJ* gene using primers CYLAT-F and CYLAT-R [29], both associated with cylindrospermopsin biosynthesis. The *sxtA* gene, associated with saxitoxin production, was amplified using primers sxtA-F and sxtA-R [30].

PCRs were performed using a DreamTaq PCR Core Kit (Thermo Scientific, Waltham, MA, USA). Each 15 µL reaction mixture contained 1.5 µL genomic DNA (10 ng µL^−1^), 1.5 µL 10× Green Buffer, 2 mM dNTP mix, 0.4 µL MgCl_2_ (25 mM), 0.075 µL DreamTaq DNA polymerase (5 U µL^−1^), and 0.3 µL (10 μM) of each forward and reverse primer. PCR cycling conditions were applied according to previously published protocols: PC-IGS amplification [27], cyrA and cyrJ [29], and sxtA [30]. The PC-IGS amplification followed [27] with minor modifications, including denaturation at 94 °C for 10 s and annealing at 47 °C for 15 s.

PCR products were visualized by electrophoresis on 1.5% TAE agarose gels, except for sxtA amplicons, which were separated on 3% agarose gels. Conventional PCR screening was used to determine the presence or absence of target genes based on band detection. Positive and negative controls were included in all PCR assays.

### 2.3. Sequencing and Phylogenetic Analysis

PCR products of the PC-IGS region were sent to BaseClear B.V. (Leiden, The Netherlands) for bidirectional Sanger sequencing. Obtained sequences were assembled and aligned using the MUSCLE algorithm implemented in Jalview version 2.11.3.0 [31].

The initial dataset consisted of PC-IGS nucleotide sequences (575 bp) from 10 strains analyzed in this study. To improve phylogenetic resolution, additional Nostocales sequences (>500 bp) were retrieved from GenBank, resulting in a final alignment of 25 sequences. Phylogenetic reconstruction was performed using the Maximum Likelihood (ML) method implemented in MEGA version 12 (v11.0.10; [32]), applying Kimura’s two-parameter substitution model with a discrete gamma distribution (+G). Node support was assessed using 1000 bootstrap replicates. Positions with less than 95% site coverage were excluded, yielding a final dataset of 495 aligned nucleotide positions.

Phylogenetic analyses were conducted on 10 morphologically representative strains to confirm species identity, whereas physiological and toxicological analyses were performed across all 39 isolates to capture intraspecific variability described below.

### 2.4. Cyanotoxin Analysis by ELISA

Microcystins (MCs) and saxitoxins (STX) were determined in 24 isolates by the enzyme-linked immunosorbent assay (ELISA) according to the manufacturers’ instruction. The Beacon Analytic Systems (Saco, ME, USA) ELISA test was used for MC analysis (a maximum detection limit of 5 µgL^−1^), and the Eurofins Abraxis (Warminster, PA, USA) ELISA test was used for STX (a maximum detection limit of 5 µg L^−1^ and 0.4 µg L^−1^, respectively). For each sample, 40 mL of culture was centrifuged at 8000× *g* for 10 min, and the resulting wet biomass was immediately frozen at −20 °C until analysis. Positive and negative controls supplied with each ELISA kit were included for quality control. Absorbance was measured at 450 nm using a LabSystems Multiskan RC microplate reader (Thermo Scientific, Waltham, MA, USA).

### 2.5. Effects of Temperature on Cyanobacterial Growth

Thirty-nine isolates of species *Aphanizomenon gracile*, *Chrysosporum bergii*, *Raphidiopsis raciborskii*, and *Sphaerospermopsis aphanizomenoides* were used for growth monoculture experiments across five temperature levels (15, 20, 25, 30, 35 °C) in 40 mL culture flasks under controlled conditions in a climate chamber (Memmert GmbH + Co. KG, Schwabach, Germany). The selected temperature range (15–25 °C) encompasses current summer water temperatures commonly observed in temperate European lakes [33], as well as elevated temperatures associated with extreme heat events and future warming scenarios (30–35 °C) [18]. Experimental treatments were maintained under the same controlled conditions as used for culture collection maintenance. Cultures were acclimated to each tested temperature for five days prior to experiments. Measurements were taken every second day for nine days using a microplate reader (Infinite 200 PRO, Tecan, Männedorf, Switzerland) with 96-well black microplates (Thermo Fisher Scientific, Rochester, NY, USA). Each culture was monitored by phycocyanin fluorescence (excitation wavelength 630 nm, emission at 660 nm), with results recorded in relative fluorescence units (RFU). RFU was used as a proxy for cyanobacterial biomass. Each experimental condition was set up in triplicate, and each fluorescence measurement was repeated five times; mean RFU values were used for growth-rate (μ) calculations. The specific growth rate (μ, day^−1^) was calculated for the exponential growth phase using the equation:

μ = ln (N_t_/N_0_)/∆t (1)

where N_0_ and N_t_ represent the mean RFU values at the beginning and end of the exponential growth phase, respectively, and ∆t is the duration of the exponential phase expressed in days [34]. We began the experiment by diluting the cultures to an initial concentration of 10 μg chlorophyll-*a* L^−1^, measured using a fluorometer (AlgaeLabAnalyser, bbe Moldaenke GmbH, Schwentinental, Germany). Biomass increase was additionally assessed based on the change in chlorophyll-*a* concentration between the first and final (10th) day of the thermotolerance experiments, using the same instrument.

### 2.6. Calculations, Data Visualization and Statistical Analysis

All statistical analyses and data visualization were performed in R (version 3.5.2), with the significance level set at *p* < 0.05. Growth rates are reported as mean ± standard deviation among replicates or isolates, as specified. Data normality was tested using the Shapiro–Wilk test; non-parametric tests were applied as assumptions were not met. Differences among groups were tested using the Kruskal–Wallis test, followed by Dunn’s post hoc test with Bonferroni correction when significant effects were detected. Overall differences in growth performance among cyanobacterial species were evaluated using permutational analysis of variance (PERMANOVA) based on Euclidean distances calculated from individual specific growth-rate (μ) values. Species identity was used as the grouping factor, and statistical significance was assessed using 999 unrestricted permutations. Homogeneity of multivariate dispersion among species was evaluated using PERMDISP based on distances to group medians, also using 999 permutations. This multivariate approach was used to test general species-level differences, whereas temperature-, isolate-, and lake-specific effects were examined using univariate non-parametric tests. Data visualization was performed using *ggplot2*, *ggpubr*, *ggpattern*, and *patchwork*.

## 3. Results

### 3.1. Morphological and Phylogenetic Analyses

Nostocales strains of *Aphanizomenon gracile*, *Chrysosporum bergii*, *Raphidiopsis raciborskii* and *Sphaerospermopsis aphanizomenoides* were identified based on both morphological features and phylogenetic analysis. Morphologically, all species, were characterised by solitary, straight trichomes with distinctive heterocytes and akinetes. *A. gracile* had cylindrical cells with aerotopes (3–5 × 3–7 μm), oval akinetes (6–8 × 12–14 μm), and spherical to oval heterocytes (5–6 × 6–7 μm) (Figure 1a–c). *C. bergii* showed barrel-shaped cells (3–4 × 4–6 μm), oval golden akinetes positioned away from heterocytes (10–13 × 15 μm), and spherical to oval heterocytes (6–8 × 5–6 μm) (Figure 1d–g). *R. raciborskii* was characterized by cylindrical cells (2–3 × 4–8 μm), long oval akinetes (5–7 × 15–17 μm), and conical terminal heterocytes (2–4 × 6–8 μm) (Figure 1h,i). *S. aphanizomenoides* displayed slightly constricted cylindrical cells (3–5 × 3–7 μm), spherical heterocytes (5–6 × 6–7 μm), and a series of round akinetes (Figure 1j–m); chytrid infection near heterocytes was commonly observed in field samples from Lake Simnas (Figure 1n).

Phylogenetic analysis using the partial PC-IGS region was presented in the maximum-likelihood shown tree that is shown in Figure 2. All investigated strains of *Aphanizomenon gracile*, *Chrysosporum bergii*, *Raphidiopsis raciborskii* and *Sphaerospermopsis aphanizomenoides* together with the corresponding GenBank sequences of the same genera formed four distinct clusters. *A. gracile* formed a monophyletic cluster with bootstrap value of 100%. The strains of *C. bergii* were clustered with a bootstrap value of 99%, although they are closely related to another species from the genus *C. ovalisporum*. *R. raciborskii* strains clustered together with *R. mediterranea* and *R. curvata* with a bootstrap value of 99%, indicating close phylogenetic relationships within the genus. Similarly, the strains of *S. aphanizomenoides* formed a separate group with a bootstrap value of 95%, which also contained sequences from *S. reniformis* and *S. eucompacta*.

### 3.2. Cyanotoxin and Cyanotoxin Biosynthesis Genes Analysis

Cyanotoxin concentrations were evaluated using a combined immunological (ELISA) and molecular (PCR) approach (Table S1). Cyanotoxins were detected in only one of the 24 Nostocales isolates analyzed. The *Aphanizomenon gracile* isolate NRC/SIM/2021/D5 was found to be positive for saxitoxins by ELISA and for the *sxtA* gene by PCR, confirming its capacity for saxitoxin production. All other isolates tested negative for cyanotoxins.

### 3.3. Thermotolerance of Native and Alien Cyanobacteria

The growth rates of the tested cyanobacterial isolates across the temperature gradient are shown in Figure 3. Overall growth performance differed significantly among species (PERMANOVA: F_3_,_565_ = 7.52, R^2^ = 0.038, *p* = 0.001). Homogeneity of dispersion was assessed using PERMDISP and was marginally significant (F_3_,_565_ = 2.57, *p* = 0.047), indicating slight heterogeneity in within-group dispersion that should be considered when interpreting the PERMANOVA results.

Temperature had a significant effect on growth rates in all species (Kruskal–Wallis test, *p* < 0.001; Table 2). Mean growth rates among isolates were similar across 20–30 °C for all species, with maximum average values recorded at 25 °C for *Raphidiopsis raciborskii* (0.42 ± 0.03 d^−1^), *Aphanizomenon gracile* (0.46 ± 0.06 d^−1^), and *Sphaerospermopsis aphanizomenoides* (0.52 ± 0.09 d^−1^), and at 30 °C for *Chrysosporum bergii* (0.46 ± 0.10 d^−1^; Figure 3). However, growth responses were species-specific at both thermal extremes (15 °C and 35 °C). At 15 °C, mean growth rates were low or negative in all alien species—*R. raciborskii*, *C. bergii*, and *S. aphanizomenoides*—whereas the native *A. gracile* maintained positive growth, indicating greater tolerance to cooler conditions. Conversely, at 35 °C, only the native *A. gracile* showed zero or negative growth, while all alien species retained positive rates (Table 3). Based on statistical results, the optimal growth interval for the native species *A. gracile* was observed at intermediate temperatures (20–30 °C; Dunn’s post hoc tests, adjusted *p* < 0.001; Table S2), while alien *C. bergii* and *S. aphanizomenoides* showed no significant differences in growth rates at temperatures ≥ 20 °C (Dunn’s post hoc test, adjusted *p* > 0.05; Table S2). *R. raciborskii* showed a similar pattern, although growth at 35 °C was slightly lower than at 25–30 °C (adjusted *p* < 0.01; Table S2).

*Aphanizomenon gracile and Chrysosporum bergii* showed significant variation among isolates (Kruskal–Wallis test, *p* < 0.05; Table 2), suggesting intraspecific heterogeneity in thermal responses. At 35 °C, some *A. gracile* isolates were completely suppressed, whereas Dunn’s post hoc tests indicated that isolates NRC/JIE/2021/C2 and NRC/GAU/2024/B7 maintained significantly higher growth rates at this temperature (Kruskal–Wallis: χ^2^ = 27.31, df = 9, *p* = 0.001; Dunn’s post hoc test, adjusted *p* < 0.05). In addition, isolate NRC/SIM/2021/D5 retained positive growth at 35 °C, indicating comparatively higher tolerance to heat stress.

*Chrysosporum bergii* was the only species showing significant differences in growth rates among isolates from different lakes (Kruskal–Wallis test, *p* < 0.05; Table 2), with growth patterns differing particularly between isolates originating from Lake Gineitiškės and Lake Jieznas (Dunn’s post hoc test, adjusted *p* < 0.001). This pattern suggests possible habitat-specific adaptation within the species.

*Raphidiopsis raciborskii* and *Sphaerospermopsis aphanizomenoides* showed no significant differences among the isolates (Kruskal–Wallis test, *p* > 0.05; Table 2). Nevertheless, *S. aphanizomenoides* exhibited considerable variability in growth rates at 20–35 °C based on standard deviation (SD = 0.10–0.11 d^−1^; Table 3), indicating higher intraspecific heterogeneity in responses to a range of temperatures.

Figure 4 shows species-specific changes in biomass over the course of the 10-day experiment, based on chlorophyll-*a* accumulation. Among the tested species, *Sphaerospermopsis aphanizomenoides* showed the highest biomass increase at all temperatures ≥ 20 °C, indicating the strongest net growth performance under warmer conditions. In comparison, other species showed more moderate biomass accumulation across the same temperature range, with *Aphanizomenon gracile* showing minimal increase at 35 °C.

## 4. Discussion

Climate modeling studies project that global mean surface temperatures will rise by approximately 1–4 °C by 2100 [35,36,37]. Heatwaves can further intensify local and short-term temperature extremes in freshwater ecosystems, with warming of up to ~2.8 °C reported in some regions [16,17,38]. Rising temperatures have major implications for freshwater ecosystems by promoting cyanobacterial blooms, especially enhancing the competitive advantage of nostocalean species [39,40,41]. Warming is also expected to facilitate the spread of non-native, warm-adapted species into new habitats across the Northern Hemisphere [12,14,15,42]. In the current study we combined phylogenetic identification, cyanotoxin-production screening, and temperature-dependent growth experiments to determine whether native and alien nostocalean cyanobacteria differ not only in taxonomic origin, but also in their potential hazard and capacity to proliferate across present and warming temperature regimes.

Phenotypic plasticity and overlapping morphology often hinder the accurate identification of nostocalean species, including alien ones [43]. Phylogenetic analysis based on the PC-IGS region, which is widely used for cyanobacterial identification and phylogenetic studies [13,26,43], helps overcome the limitations of morphology-based identification. Following a polyphasic approach, isolates were initially identified according to morphological characteristics, including key features such as trichome structure, heterocyte form and position, akinete morphology, and cell dimensions. The identification of *Aphanizomenon gracile*, *Chrysosporum bergii*, *Raphidiopsis raciborskii*, and *Sphaerospermopsis aphanizomenoides* was subsequently confirmed based on partial PC-IGS sequences (Figure 2). It provides confidence that the isolates identified using the same morphological features are correctly assigned at the species level, thereby supporting subsequent analyses of their toxicological and physiological traits.

One key factor influencing the establishment and competitive success of cyanobacteria in new aquatic environments is their ability to produce allelochemicals, including cyanotoxins, which can affect phytoplankton succession and bloom formation [44]. In the present study, for the native species *Aphanizomenon gracile* one isolate (NRC/SIM/2021/D5) out of five tested was found to possess the *stxA* gene, and saxitoxin production was confirmed by ELISA (Table S1). This finding agrees with Karosienė [13], who reported only 17% of saxitoxin-producing *A. gracile* isolates in the population. *Chrysosporum bergii* is known as a potential producer of cylindrospermopsin in Australia [45], whereas *Raphidiopsis raciborskii* has been identified as a producer of cylindrospermopsin and saxitoxin in South America and Australia [46]. In addition, unconfirmed cyanotoxin production by *Sphaerospermopsis aphanizomenoides* has been reported in Africa [47]. However, our results are consistent with previous studies [12,48,49] suggesting that those species that are alien to Europe are unlikely to be cyanotoxin producers in the continent.

Environmental change may facilitate the spread of alien cyanobacteria while simultaneously increasing parasite pressure, as temperature is a key abiotic factor shaping parasite–host interactions [50]. In this study, we observed chytrid fungal infection of *Sphaerospermopsis aphanizomenoides* heterocytes in field samples from Lake Simnas (Figure 1). Chytrid parasitism is known to reduce cyanobacterial fitness and influence bloom dynamics, acting as a natural top-down control mechanism in freshwater ecosystems [51]. Notably, *S. aphanizomenoides* accounted for 27% of total cyanobacterial biomass in Lake Simnas in July but was short-lived [52], suggesting that parasitism may have constrained its persistence. Such interactions support the idea that alien cyanobacteria may be susceptible to native parasitic fungi, which could limit their establishment and ecological dominance [53].

### Thermal Growth Response of Alien and Native Cyanobacteria from Temperate Freshwaters

In this study, we investigated the thermal responses of 39 native and alien cyanobacterial isolates originating from freshwater ecosystems in the temperate climate zone of Central and Northern Europe (Lithuania, Poland, and the Czech Republic) (Table 1). Mean surface water temperatures during the growth season in this region typically range from 15 to 25 °C, while summer maxima in shallow lakes and reservoirs frequently reach 20–25 °C [15,33], reflecting current climatic conditions. Long-term observations and climate projections indicate further increases in summer temperatures across this region, with heatwaves expected to intensify thermal extremes in freshwater ecosystems [18,54]. Therefore, growth experiments conducted at 30 °C and 35 °C were designed to simulate plausible future warming scenarios and assess the thermal tolerance of cyanobacteria beyond current mean environmental temperatures.

The native species *Aphanizomenon gracile* and the alien species *Chrysosporum bergii*, *Raphidiopsis raciborskii*, and *Sphaerospermopsis aphanizomenoides*, which co-occur in European freshwaters [52], showed distinct temperature-dependent growth patterns in the present study. Although all species grew well at moderate temperatures, their responses diverged at thermal extremes. The native species *A. gracile* performed best at intermediate temperatures (20–30 °C) and was the only species capable of sustaining growth at 15 °C (Figure 3; Table 3). In contrast, the alien cyanobacterial species showed similar thermal responses but had higher temperature optima, ranging from 20 to 35 °C, indicating broad thermal tolerance under warm conditions. At 15 °C, however, the growth rates of all alien species were low or negative, reflecting strong physiological constraints at lower temperatures (Figure 3; Table 3).

In our study, *Aphanizomenon gracile* showed the highest mean growth rate across isolates at 25 °C (0.46 ± 0.06 d^−1^; Table 3), consistent with the reported optima of other Lithuanian isolates at 23–28 °C (~0.4–0.5 d^−1^; [15]). Similarly, a German isolate reached its maximum growth rate at 28 °C (0.29 d^−1^; [14]), whereas substantially higher rates were reported for a Dutch isolate at elevated temperatures (0.87 d^−1^ at 32.5 °C and 0.85 d^−1^ at 35 °C). These discrepancies likely reflect methodological differences among studies, including the range and resolution of temperature treatments. In our study, *A. gracile* also showed increased variability among isolates at 35 °C (Figure 3; Table 2): while some isolates were strongly inhibited, others maintained positive growth, indicating considerable intraspecific variation in heat tolerance. This finding suggests that the thermal responses of *A. gracile* populations under climate warming may depend more on local strain composition than on uniform species-level traits.

A key ecological characteristic distinguishing *Aphanizomenon gracile* from the alien species is its ability to sustain growth under cooler conditions. In our experiment, *A. gracile* maintained positive mean growth at 15 °C (0.25 d^−1^), consistent with previous reports of growth at 10–15 °C (~0.15–0.20 d^−1^; [14]). This species can initiate growth at temperatures that effectively preclude active growth of alien cyanobacteria, enabling it to proliferate in early spring and late autumn when water temperatures remain too low for its competitors. An experimental study by Savadova et al. [42] further demonstrated that native *A. gracile* could suppress both native (*Planktothrix agardhii*) and alien (*Chrysosporum bergii* and *Sphaerospermopsis aphanizomenoides*) species under moderate temperatures (20 °C). Although the alien species became more competitive under elevated temperatures and nutrient enrichment, they did not completely outcompete *A. gracile*, possibly because the highest temperature tested (24 °C) was insufficient to promote strong alien dominance. This thermal advantage positions *A. gracile* as a temperate-adapted species with a distinct seasonal niche during cooler periods.

Among the alien species, *Chrysosporum bergii* showed a maximum mean growth rate of 0.46 ± 0.10 d^−1^ at 30 °C (Table 3), suggesting that climate warming may enhance its proliferation. Our results are consistent with growth rates of 0.32–0.53 d^−1^ reported at 26–30 °C by Savadova et al. [15]. In agreement with our findings (Figure 3; Table 3), Mehnert et al. [14] observed low growth rates at 15 °C (~0.1 d^−1^) and negative growth at 10 °C, confirming the limited performance of *C. bergii* under cooler conditions. However, they reported a lower maximum growth rate for a German isolate at 26.5 °C (0.25 d^−1^), indicating substantial variability among strains. In our study, *C. bergii* demonstrated considerable intraspecific variation among isolates originating from different lakes (Table 2), suggesting that the thermal performance of *C. bergii* is influenced by local habitat conditions. Although *C. bergii* is frequently recorded in temperate regions where summer water temperatures remain moderate, bloom formation has not been reported [12]. Additionally, *C. bergii’s* limited bloom formation in Europe may be related to its preference for elevated salinity, consistent with its Ponto–Caspian origin [8,12,55]. Therefore, elevated temperatures could enhance its growth performance and potentially increase its bloom-forming capacity, especially when combined with other favorable environmental conditions.

In our study, the highest mean growth rate of the alien cyanobacterium *Sphaerospermopsis aphanizomenoides* (0.52 ± 0.09 d^−1^) was recorded at 25 °C, which is lower than the optimum temperature of 30 °C (0.36 d^−1^) reported by Mehnert et al. [14]. Savadova et al. [15] showed that two Lithuanian isolates performed similarly between 22 and 30 °C but diverged markedly at 35 °C, with one isolate reaching 0.70 d^−1^ while the other declined to 0.32 d^−1^. These findings confirm that pronounced isolate-specific variability occurs in *S. aphanizomenoides*, as observed in our experiment as well. Consistent with Mehnert et al. [14], our study found low or negative growth at 10–15 °C (Figure 3; Table 3), indicating limited tolerance to cooler conditions. Field observations further support this temperature-dependent pattern, as blooms near the species’ northern distribution limit are typically associated with elevated water temperatures (~30 °C), whereas under cooler conditions *S. aphanizomenoides* generally remains at low abundance [56,57]. While growth rates among alien cyanobacteria were broadly similar (Figure 3), *S. aphanizomenoides* showed the highest cumulative biomass increase across all temperatures ≥ 20 °C (Figure 4). This discrepancy suggests that *S. aphanizomenoides* may sustain exponential growth for longer, accumulate more chlorophyll-*a*, or possess morphological traits that enhance pigment production. Ecologically, such sustained biomass accumulation under warm conditions could provide a competitive advantage by enabling *S. aphanizomenoides* to dominate in nutrient-rich environments and outcompete slower-accumulating species during extended warm periods.

The highest mean growth rate of *Raphidiopsis raciborskii* was recorded at 25 °C (0.42 ± 0.03 d^−1^). Previous studies similarly identified 20–35 °C as the optimal temperature range, with maximum growth typically occurring near 30 °C [58]. Mehnert et al. [14] reported an optimum at 28 °C (0.34 d^−1^) for a German isolate, whereas a Dutch isolate achieved substantially higher growth rates at similar temperatures (0.81 d^−1^; [59]), highlighting considerable variation among isolates in Europe. Field observations from temperate lakes indicate that *R. raciborskii* can dominate phytoplankton communities at temperatures around 17 °C [58], although blooms occur more frequently during unusually warm summers [60,61]. Experimental studies have shown that *R. raciborskii* can maintain biomass at relatively low temperatures (~17 °C) within established phytoplankton communities [62]. In contrast, our monoculture experiments, consistent with Mehnert et al. [14], revealed low or negative growth at 10–15 °C (Figure 3; Table 3). Although *R. raciborskii* has been recorded in Lake Jieznas in Lithuania since 1988, it has never been reported to form blooms, likely due to insufficient summer temperatures [12,63].

Although our study did not detect significant intraspecific variation in the growth rates of *Raphidiopsis raciborskii*, substantial evidence indicates the existence of distinct ecotypes within this species, accompanied by considerable physiological and genomic diversity among strains [58,64,65]. Strains from temperate regions tolerate low temperatures better than tropical or subtropical strains, indicating geographically structured thermal adaptations often described as cold-tolerant ecotypes [5,66]. These findings suggest that the expansion of *R. raciborskii* into temperate regions is driven by pre-existing strain-level thermal diversity rather than short-term phenotypic plasticity alone. Recent studies further indicate that invasion success depends not only on temperature but also on interacting environmental factors, including nitrogen availability and thermal stability [67,68,69]. Although temperate strains can establish and maintain biomass under cool conditions, they often fail to dominate under fluctuating temperatures or nutrient-limited regimes, highlighting the importance of strain-specific traits and ecological context [68]. Together, these findings underscore the critical role of strain-level variation in shaping the distribution and bloom dynamics of *R. raciborskii*. Although all alien species in this study showed enhanced growth under elevated temperatures, *Raphidiopsis raciborskii* remains the most widespread and extensively studied alien cyanobacterium in Europe.

Ongoing climate warming is expected to shift competitive balances in temperate freshwaters toward warm-adapted alien cyanobacteria, potentially increasing their dominance and bloom intensity. However, our results show that under current temperature regimes, native and alien species can show comparable growth performance under laboratory conditions, indicating that temperature alone does not fully explain invasion success. Increasing evidence highlights the importance of interacting drivers, including strain-level variability, nutrient levels, phenotypic plasticity, and thermal acclimation, which can strongly modulate growth responses [20,70,71]. In addition, anthropogenic habitat modification—particularly eutrophication and hydrological alteration—has been repeatedly linked to enhanced invasion risk [72,73,74]. Consistent with this pattern, all alien cyanobacteria in our study occurred in urbanized eutrophic or hypereutrophic systems (Table 1), indicating that such environments are particularly susceptible to the establishment of alien species and, under ongoing environmental change, may be prone to their rapid proliferation and dominance.

## 5. Conclusions

This study reveals both interspecific and intraspecific differences in temperature-driven growth responses among native and alien nostocalean cyanobacteria in temperate European freshwaters. The native *Aphanizomenon gracile* maintained growth at cooler temperatures, reflecting its adaptation to temperate climates. In contrast, alien species—*Chrysosporum bergii*, *Raphidiopsis raciborskii*, and *Sphaerospermopsis aphanizomenoides*—showed broader thermal tolerance under warm conditions but were limited at lower temperatures. Although native and alien taxa performed similarly under current thermal regimes, future warming is likely to reduce the seasonal advantage of cold-adapted natives and favor the expansion of warm-adapted alien species.

Among the four species screened, cyanotoxins production was detected only in the native species *A. gracile*. However, their ability to thrive under elevated temperatures raises concerns about the potential introduction or proliferation of toxigenic genotypes reported in other continents.

High variability among isolates, especially at thermal extremes, suggests that responses to warming depend on local strain composition rather than species-wide characteristics. This underscores the importance of including multiple strains in experimental studies to capture intraspecific diversity. Understanding future phytoplankton community dynamics in temperate lakes will require combined assessments of thermal tolerance, strain-level traits, response to nutrient levels, habitat changes, and ongoing environmental change.

## Figures and Tables

**Figure 1:**
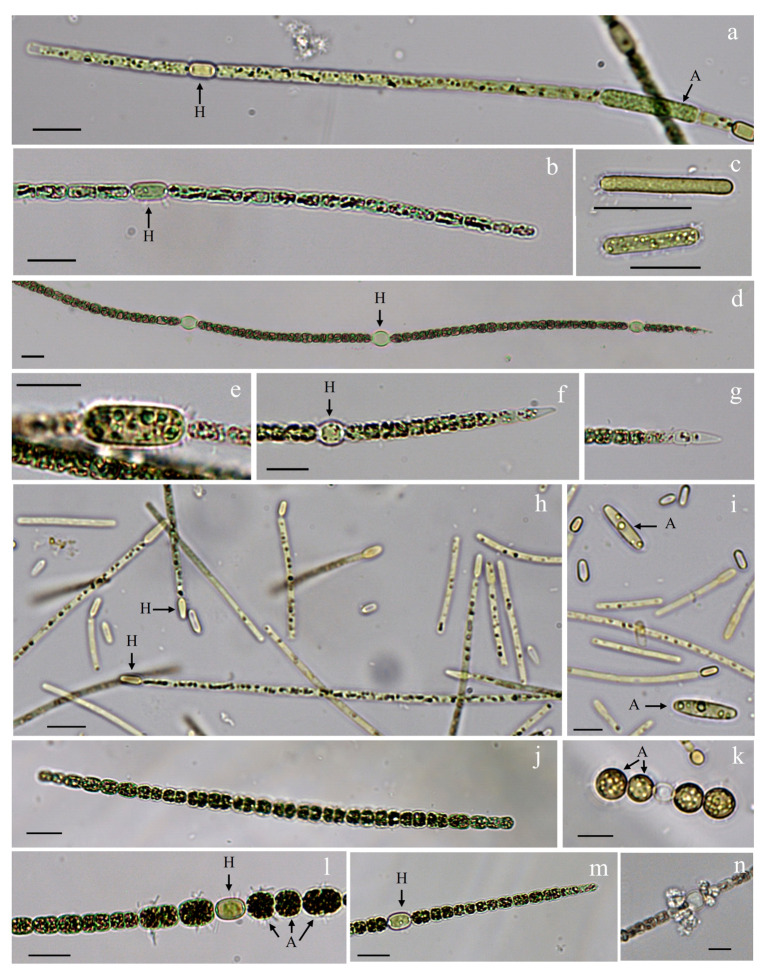
Microscopic images of the studied cyanobacterial isolates. (**a**–**c**)—*Aphanizomenon gracile* ((**a**)—filament with heterocytes and typical terminal cells; (**c**)—akinetes); (**d**–**g**)—*Chrysosporum bergii* ((**d**)—typical filament; (**e**)—newly formed akinete; (**f**)—filament with a heterocyte; (**g**)—typical terminal cell); (**h**,**i**)—*Raphidiopsis raciborskii* ((**h**)—filaments with terminal heterocytes; (**i**)—akinetes); (**j**–**n**)—*Sphaerospermopsis aphanizomenoides* ((**j**)—filament; (**k**)—mature akinetes; (**l**)—filament with a heterocyte and newly formed akinetes; (**m**)—filament with a heterocyte; (**n**)—*S. aphanizomenoides* from Lake Simnas field samples infected by chytrid fungi). Abbreviations: H—heterocyte; A—akinete. Scale bar in all images—10 µm.

**Figure 2:**
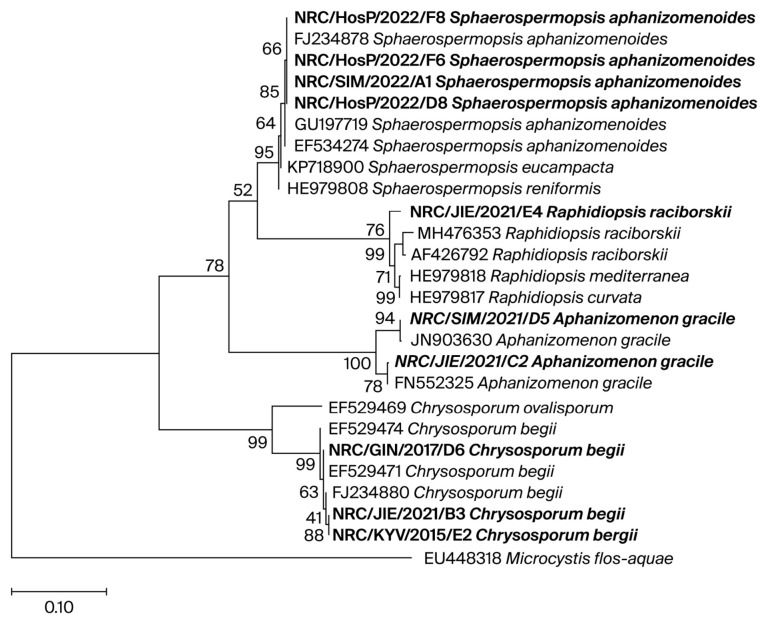
Maximum likelihood tree based on partial PC-IGS region sequences of 25 Nostocales strains. Strains used in this study are shown in bold; reference sequences retrieved from GenBank are indicated by accession numbers. Bootstrap support values are shown at the nodes. The scale bar represents the number of substitutions per site. *Microcystis flos-aquae* (GenBank accession no. EU448318) was used as the outgroup.

**Figure 3:**
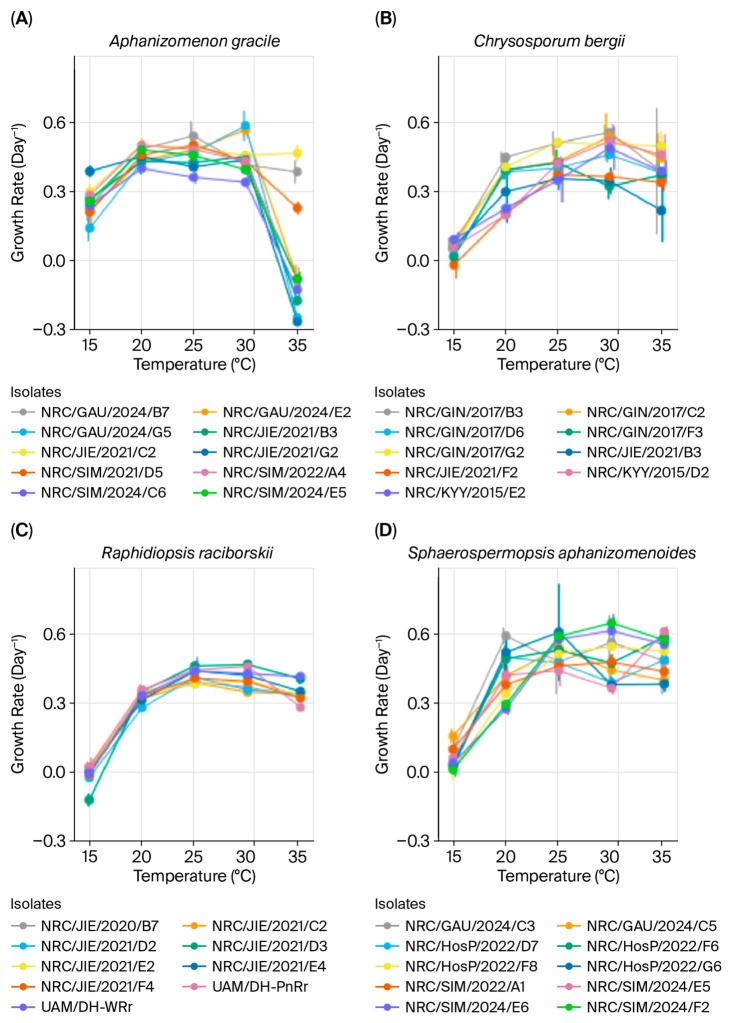
Growth rates of cyanobacterial isolates across a range of different temperatures. Panels show the four studied species: (**A**) *Aphanizomenon gracile*, (**B**) *Chrysosporum bergii*, (**C**) *Raphidiopsis raciborskii*, and (**D**) *Sphaerospermopsis aphanizomenoides*. Vertical bars represent the standard deviation of the mean growth rate.

**Figure 4:**
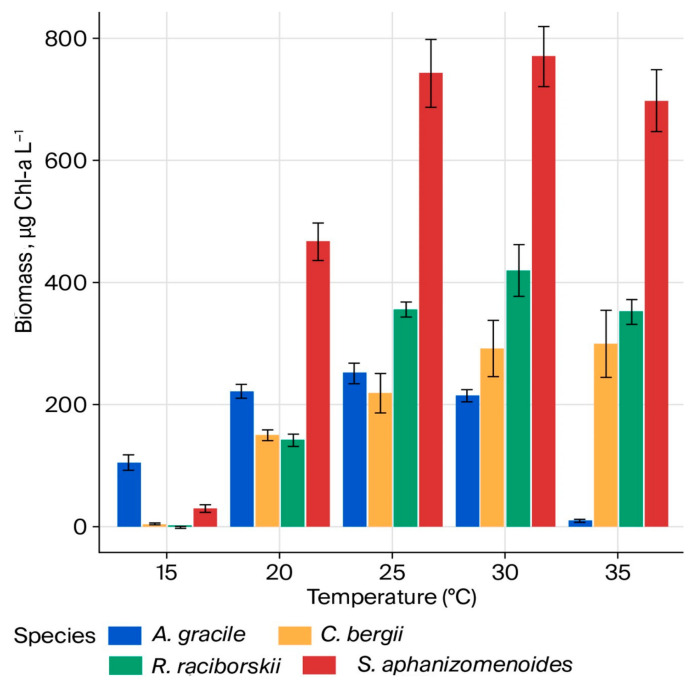
Mean biomass (µg Chl-*a* L^−1^) of *Aphanizomenon gracile*, *Chrysosporum bergii*, *Raphidiopsis raciborskii*, and *Sphaerospermopsis aphanizomenoides* at different temperatures over the experiment.

**Table 1:** List of the studied cyanobacterial isolates, including their origin, year of isolation, habitat conditions, and the analyses performed.

Isolate	Water Body	Year of Isolation	Trophic Status of Lake	Assessment		
				Phylogeny Assessment	Cyanotoxin Screening	Thermotolerance
*Aphanizomenon gracile*						
NRC/SIM/2022/A4	Lake Simnas	2022	Hypertrophic ^a^		x	X
NRC/SIM/2021/D5	Lake Simnas	2021	Hypertrophic ^a^	x	x	X
NRC/SIM/2024/C6	Lake Simnas	2024	Hypertrophic ^a^			X
NRC/SIM/2024/E5	Lake Simnas	2024	Hypertrophic ^a^			X
NRC/JIE/2021/B3	Lake Jieznas	2021	Eutrophic ^a^		x	X
NRC/JIE/2021/C2	Lake Jieznas	2021	Eutrophic ^a^	x	x	X
NRC/JIE/2021/G2	Lake Jieznas	2021	Eutrophic ^a^		x	X
NRC/GAU/2024/B7	Lake Gauštvinis	2024	Hypertrophic ^b^			X
NRC/GAU/2024/G5	Lake Gauštvinis	2024	Hypertrophic ^b^			X
NRC/GAU/2024/E2	Lake Gauštvinis	2024	Hypertrophic ^b^			X
*Chrysosporum bergii*						
NRC/KYV/2015/D2	Lake Rėkyva	2015	Eutrophic ^d^		x	X
NRC/KYV/2015/E2	Lake Rėkyva	2015	Eutrophic ^d^	x	x	X
NRC/GIN/2017/B3	Lake Gineitiškės	2017	Hypertrophic ^a^		x	X
NRC/GIN/2017/C2	Lake Gineitiškės	2017	Hypertrophic ^a^		x	X
NRC/GIN/2017/D6	Lake Gineitiškės	2017	Hypertrophic ^a^	x	x	X
NRC/GIN/2017/F3	Lake Gineitiškės	2017	Hypertrophic ^a^		x	X
NRC/GIN/2017/G2	Lake Gineitiškės	2017	Hypertrophic ^a^		x	X
NRC/JIE/2021/B3	Lake Jieznas	2021	Eutrophic ^a^	x	x	X
NRC/JIE/2021/F2	Lake Jieznas	2021	Eutrophic ^a^		x	X
*Raphidiopsis raciborskii*						
NRC/JIE/2020/B7	Lake Jieznas	2021	Eutrophic ^a^			X
NRC/JIE/2021/C2	Lake Jieznas	2021	Eutrophic ^a^		x	X
NRC/JIE/2021/D2	Lake Jieznas	2021	Eutrophic ^a^		x	X
NRC/JIE/2021/D3	Lake Jieznas	2021	Eutrophic ^a^		x	X
NRC/JIE/2021/E2	Lake Jieznas	2021	Eutrophic ^a^		x	X
NRC/JIE/2021/E4	Lake Jieznas	2021	Eutrophic ^a^	x	x	X
NRC/JIE/2021/F4	Lake Jieznas	2021	Eutrophic ^a^		x	X
UAM/DH-PnRr	Lake Pniewskie	2017	Hypertrophic ^c^			X
UAM/DH-WRr	Lake Witobelskie	2017	Hypertrophic ^c^			X
*Sphaerospermopsis aphanizomenoides*						
NRC/SIM/2022/A1	Lake Simnas	2022	Hypertrophic ^a^	x	x	X
NRC/SIM/2024/E5	Lake Simnas	2024	Hypertrophic ^a^			X
NRC/SIM/2024/E6	Lake Simnas	2024	Hypertrophic ^a^			X
NRC/SIM/2024/F2	Lake Simnas	2024	Hypertrophic ^a^			X
NRC/HosP/2022/D7	Reservoir Hostivař	2022	-			X
NRC/HosP/2022/D8	Reservoir Hostivař	2022	-	x	x	
NRC/HosP/2022/F6	Reservoir Hostivař	2022	-	x	x	X
NRC/HosP/2022/F8	Reservoir Hostivař	2022	-	x	x	X
NRC/HosP/2022/G6	Reservoir Hostivař	2022	-			X
NRC/GAU/2024/C3	Lake Gauštvinis	2024	Hypertrophic ^a^			X
NRC/GAU/2024/C5	Lake Gauštvinis	2024	Hypertrophic ^a^			X

^a^—[12], ^b^—[24], ^c^—[4], ^d^—[25].

**Table 2:** Results of the Kruskal–Wallis test (χ^2^) evaluating the differences in growth rates under various temperatures and among strains of cyanobacteria. Bold *p*-values indicate statistically significant differences (*p* < 0.05).

Species	Factor	χ^2^	df	*p*-Value
*Aphanizomenon gracile*	Isolates	17.31	9	**0.044**
	Temperature	91.94	4	**<0.001**
	Lake	2.51	2	0.285
*Chrysosporum bergii*	Isolates	20.65	8	**0.008**
	Temperature	73.63	4	**<0.001**
	Lake	13.40	2	**0.001**
*Raphidiopsis raciborskii*	Isolates	9.28	8	0.319
	Temperature	101.01	4	**<0.001**
	Lake	2.72	2	0.256
*Sphaerospermopsis aphanizomenoides*	Isolates	7.92	9	0.542
	Temperature	80.27	4	**<0.001**
	Lake	0.29	2	0.867

**Table 3:** Mean (± SD) specific growth rates (d^−1^) of cyanobacterial isolates across temperatures.

Temperature, °C	*Aphanizomenon gracile*	*Chrysosporum bergii*	*Raphidiopsis raciborskii*	*Sphaerospermopsis aphanizomenoides*
15	0.25 ± 0.07	0.05 ± 0.05	−0.01 ± 0.05	0.06 ± 0.05
20	0.46 ± 0.04	0.33 ± 0.10	0.33 ± 0.03	0.42 ± 0.11
25	0.46 ± 0.06	0.42 ± 0.07	0.42 ± 0.03	0.52 ± 0.09
30	0.45 ± 0.08	0.46 ± 0.10	0.40 ± 0.04	0.49 ± 0.11
35	0.00 ± 0.26	0.39 ± 0.13	0.35 ± 0.04	0.50 ± 0.09

## Data Availability

The original contributions presented in this study are included in the article/Supplementary Materials. Further inquiries can be directed to the corresponding authors.

## Supplementary Materials

The following supporting information can be downloaded at https://www.mdpi.com/article/10.3390/biology15151316/s1, Table S1: Cyanotoxins and cyanotoxin biosynthesis genes detected in the Nostocales strains. Table S2: Pairwise comparisons of specific growth rates among temperatures obtained using Dunn’s post hoc test with Bonferroni correction. Table S3: Pairwise comparisons of specific growth rates among *Aphanizomenon gracile* isolates at 35 °C obtained using Dunn’s post hoc test with Bonferroni correction.
