# Supplementary material for: Phylogenetic Identity, Cyanotoxin Production Potential, and Thermal Performance in Native and Alien Nostocalean Cyanobacteria Under Climate Warming
Source: Biology (Basel). 2026 Aug 6;15(15):1316. doi: 10.3390/biology15151316 (PMC13464402; doi:10.3390/biology15151316)
Supplement: Supplementary file 1 [file biology-15-01316-s001.zip › biology-4444791-Supplementary file.pdf]

Supplementary file

**Table S1.** Cyanotoxins and cyanotoxin biosynthesis genes detected in the Nostocales strains.

| **Cyanotoxins** | **CYN** | | **MCs** | **SXT** | |
|---|---|---|---|---|---|
| **Assessment** | PCR | | ELISA, μg $L^{-1}$ | PCR | ELISA, μg $L^{-1}$ |
| **Starins** | *cyrA* | *cyrJ* | | *sxtA* | |
| ***Aphanizomenon gracile*** | | | | | |
| NRC/SIM/2022/A4 | - | - | | - | |
| NRC/SIM/2021/D5 | - | - | | + | + |
| NRC/JIE/2022/B3 | - | - | | - | |
| NRC/JIE/2022/C2 | - | - | | - | |
| NRC/JIE/2022/G2 | - | - | | - | |
| ***Chrysosporum bergii*** | | | | | |
| NRC/KYV/2015/D2 | - | - | | - | |
| NRC/KYV/2015/E2 | - | - | | - | |
| NRC/GIN/2017/B3 | - | - | | - | |
| NRC/GIN/2017/C2 | - | - | | - | |
| NRC/GIN/2017/D6 | - | - | | - | |
| NRC/GIN/2017/F3 | - | - | | - | |
| NRC/GIN/2017/G2 | - | - | | - | |
| NRC/JIE/2021/B3 | - | - | | - | |
| NRC/JIE/2021/F2 | - | - | | - | |
| ***Raphidiopsis raciborskii*** | | | | | |
| NRC/JIE/2021/C2 | - | - | | - | |
| NRC/JIE/2021/D2 | - | - | | - | |
| NRC/JIE/2021/D3 | - | - | | - | |
| NRC/JIE/2021/E2 | - | - | | - | |
| NRC/JIE/2021/E4 | - | - | | - | |
| NRC/JIE/2021/F4 | - | - | | - | |
| ***Sphaerospermopsis aphanizomenoides*** | | | | | |
| NRC/SIM/2022/A1 | - | - | - | - | |
| NRC/HosP/2022/D8 | - | - | - | - | |
| NRC/HosP/2022/F6 | - | - | - | - | |
| NRC/HosP/2022/F8 | - | - | - | - | |

**Table S2.** Pairwise comparisons of specific growth rates among temperatures obtained using Dunn's post-hoc test with Bonferroni correction.

| **Species** | **Comparison** | **Z** | **Adjusted p** |
|---|---|---|---|
| *Aphanizomenon gracile* | 15–20 | -5.860 | **<0.001** |
| | 15–25 | -5.988 | **<0.001** |

| | | | |
|---|---|---|---|
| | 20–25 | -0.128 | 1.000 |
| | 15–30 | -5.046 | **<0.001** |
| | 20–30 | 0.814 | 1.000 |
| | 25–30 | 0.942 | 1.000 |
| | 15–35 | 0.981 | 1.000 |
| | 20–35 | 6.840 | **<0.001** |
| | 25–35 | 6.968 | **<0.001** |
| | 30–35 | 6.026 | **<0.001** |
| *Chrysosporum bergii* | 15–20 | -4.665 | **<0.001** |
| | 15–25 | -6.863 | **<0.001** |
| | 20–25 | -2.198 | 0.280 |
| | 15–30 | -7.739 | **<0.001** |
| | 20–30 | -3.074 | 0.021 |
| | 25–30 | -0.876 | 1.000 |
| | 15–35 | -5.813 | **<0.001** |
| | 20–35 | -1.192 | 1.000 |
| | 25–35 | 0.984 | 1.000 |
| | 30–35 | 1.852 | 0.640 |
| *Sphaerospermopsis aphanizomenoides* | 15–20 | -4.986 | **<0.001** |
| | 15–25 | -7.678 | **<0.001** |
| | 20–25 | -2.692 | 0.071 |
| | 15–30 | -6.686 | **<0.001** |
| | 20–30 | -1.700 | 0.892 |
| | 25–30 | 0.992 | 1.000 |
| | 15–35 | -7.393 | **<0.001** |
| | 20–35 | -2.407 | 0.161 |
| | 25–35 | 0.285 | 1.000 |
| | 30–35 | -0.707 | 1.000 |
| *Raphidiopsis raciborskii* | 15–20 | -3.848 | **0.001** |
| | 15–25 | -8.830 | **<0.001** |
| | 20–25 | -4.982 | **<0.001** |
| | 15–30 | -8.064 | **<0.001** |
| | 20–30 | -4.217 | **<0.001** |
| | 25–30 | 0.765 | 1.000 |
| | 15–35 | -4.620 | **<0.001** |
| | 20–35 | -0.772 | 1.000 |
| | 25–35 | 4.210 | **<0.001** |
| | 30–35 | 3.444 | **0.006** |

**Table S3.** Significant pairwise comparisons of specific growth rates among *Aphanizomenon gracile* isolates at 35 °C, based on Dunn's post-hoc test with Bonferroni correction.

| Comparison | Z | Adjusted *p* |
|---|---|---|
| NRC/GAU/2024/G5 – NRC/JIE/2021/C2 | −3.34 | 0.038 |
| NRC/GAU/2024/B7 – NRC/JIE/2021/G2 | 3.34 | 0.038 |
| NRC/JIE/2021/C2 – NRC/JIE/2021/G2 | 3.76 | 0.008 |
